# IL-21 promotes osteoblastic differentiation of human valvular interstitial cells through the JAK3/STAT3 pathway

**DOI:** 10.7150/ijms.49533

**Published:** 2020-10-20

**Authors:** Zongtao Liu, Yixuan Wang, Jiawei Shi, Si Chen, Li Xu, Fei Li, Nianguo Dong

**Affiliations:** Department of Cardiovascular Surgery, Wuhan Union Hospital, Huazhong University of Science and Technology, Wuhan, China.

**Keywords:** calcific aortic valve disease, Valvular interstitial cells, IL-21, valvular calcification, JAK3, STAT3

## Abstract

**Objectives**: This study amied to whether IL-21 promotes osteoblast transdifferentiation of cultured human Valvular interstitial cells (VICs).

**Methods**: We first confirmed that IL-21 alters gene expression between CAVD aortic valve tissue and normal samples by immunohistochemistry, qPCR, and western blotting. VICs were cultured and treated with IL-21. Gene and protein expression levels of the osteoblastic markers ALP and Runx2, which can be blocked by specific JAK3 inhibitors and/or siRNA of STAT3, were measured.

**Results:** IL-21 expression was upregulated in calcified aortic valves and promotes osteogenic differentiation of human VICs. IL-21 accelerated VIC calcification through the JAK3/STAT3 pathway.

**Conclusion:** Our data suggest that IL-21 is a key factor in valve calcification and a promising candidate for targeted therapeutics for CAVD.

## Introduction

Calcific aortic valve disease (CAVD) is a heart disease common in the elderly characterized by valvular calcification, fibrosis and inflammation. 1, 2. It is a major public health problem in developed countries 3. To date, no drugs have effectively prevented or altered the course of this disease and aortic valve replacement is the only effective treatment 4. Although the pathologic mechanism of CAVD remains incompletely understood, it has been reported that CAVD is an active disease with multiple pathological changes that are similar to vascular calcification, including inflammation, osteogenesis, and mineralization 5. Inflammation appears to play an essential role in the development of aortic valve leaflet calcification on the basis of pathology 6-8.

Valvular interstitial cells (VICs) and valvular endothelial cells are the most important cellular components of natural valves. There is abundant evidence that VICs play an important role in valvular calcification 9. Meanwhile, VICs are heterogeneous group of fibroblasts 10. When activated by pathological factors such as pro-inflammatory cytokines, mechanical stress, and changes in extracellular matrix composition, VICs can transform into myofibroblasts or osteoblast-like cells 10,11. Multiple studies indicate that cytokines such as interleukin-6, as well as tumor necrosis factor-a (TNF-a), IL18, and HMGB-1 induce differentiation and mineralization of VICs 11, 12.

IL-21 is an inflammatory cytokine that belongs to the IL-2 class of cytokines. After binding to the IL-21 receptor (IL-21R) 13, the JAK/STAT signaling pathway is activated to induce inflammation. IL-21R is expressed primarily by immune cells, including T cells and natural killer (NK) cells. Notably, IL-21R is strongly expressed in rheumatoid arthritis-fibroblast-like synoviocytes (RA-FLSs) in rheumatoid synovial tissue 14, 15. The IL-21/IL-21R axis contributes to the pathogenesis of RA-FLSs through activation of the JAK/STAT signaling pathway 16. Interleukin-6 (IL-6)-activated JAK/STAT signaling induces osteogenic differentiation and mineralization of VICs. However, it is not known whether IL-21 promotes the osteoblastic differentiation of VICs. Therefore, we investigated the stimulatory effects of IL-21 on osteoblastic differentiation of VICs.

## Materials and Methods

### Human aortic valve collection

This study complied with the Declaration of Helsinki and was approved by the review boards of Union Hospital and Tongji Medical College (approval IORG0003571). A total of 20 patients with CAVD and 15 control patients who underwent heart transplantation were enrolled at Union Hospital from 2017 to 2019. All patients provided informed consent for the use of their clinical specimens for research. Patients with congenital aortic valve abnormality, rheumatic disease, or endocarditis were excluded. Demographic information is summarized in Supplementary Table 1. Tissue samples were kept frozen in liquid nitrogen until use. Their paraffin-embedded aortic valve tissue samples were collected for immunohistochemistry.

### Cell culture and treatments

Human VICs were isolated and characterized as previously described 17. Passages 3-5 were used in all experiments. Cells were cultured in DMEM containing 10% fetal bovine serum (FBS; Gibco, New Zealand), in a humidified incubator at 37 °C. When they reached 70-80% confluency, the medium was changed to DMEM containing only 2% FBS, the cells were cultured overnight, and treated with recombinant human IL-21 (PeproTech, USA). Other pharmacologic reagents, including 10 mmol/L WHI-P154 (purchased from Selleck Chemicals, USA), were added 2 h before the addition of IL-21. Cells from 3 patients were used for each intervention.

### Western blotting

Total protein and nuclear protein lysates were extracted from cultured VICs using commercial buffers (Thermo Fisher, USA) according to the manufacturers' instructions. The primary antibodies used in this study were anti-Runx2 (ab23981, 1:500, Abcam, USA) and anti-IL-21 (ab119542, 1:1000, Abcam) along with anti-STAT3 (#4695, 1:1000, Cell Signaling Technology, USA) and anti-ALP (#8480, 1:1000 Cell Signaling Technology). Anti- GAPDH (60004-1-Ig, 1:5000) was purchased from Proteintech (China). Primary antibodies were incubated overnight at 4 °C. Blots were washed three times for 15 min with Tris-buffered saline with 0.1% Tween-20 (TBST) followed by incubation for 2 h at room temperature with secondary antibodies. After three washes for 15 minutes with TBST, specific staining was detected using chemiluminescence (ECL) system. All bands were densitometrically analysed with ImageJ.

### Quantitative PCR (qPCR)

Total RNA was extracted from VICs and human tissue samples with Trizol (Ambion, Carlsbad, CA) reagent according to the manufacturer's instructions. Reverse transcription of cDNAs was performed from 1 ug of isolated total RNA using Superscript II Reverse Transcriptase (Invitrogen, Carlsbad, CA). PCR reactions were performed with 40 cycles of amplification with an ABI StepOnePlus real‐time PCR system (Applied Biosystems, USA). Primers are listed in Supplementary Table 2. Data were evaluated by the 2^-ΔΔCt^ method.

### Alizarin Red S staining

For mineralization experiments, VICs were seeded in 24-well plates. At 80% confluence, cells were incubated, per indicated interventions, in medium for 21 days. The medium was exchanged every 3 days during this culture period. Alizarin Red S staining was then conducted using a commercial Alizarin Red S staining kit (ScienCell #0223, USA).

### Immunohistochemistry

Valve tissue samples were fixed with 4% paraformaldehyde and embedded in paraffin. Immunohistochemistry was performed to detect Alizarin Red S and IL-21 (ab5978, 1:100). Briefly, after dehydration, sections were boiled for 2 min in citrate buffer (pH 6.0) for antigen retrieval, followed by treatment with 3% H_2_O_2_ to block endogenous peroxidase activity. Subsequently, the sections were incubated overnight at 4 ºC with the primary antibodies, followed by incubation with an appropriate second antibody for 30 min at room temperature. DAB was used as a substrate for staining.

### Immunofluorescence staining

Cells in the samples were collected and washed twice in PBS before being fixed in 2 mL 4% paraformaldehyde for 10min.After that,the samples were washed twice in PBS and permeabilized by incubation with 2 mL of 0.1% Triton X-100 in PBS for 15 min. Then, after washing thrice with PBS and blocking for 1 h, Cells were incubated with IL-21R (ab5980, diluted 1:100), antibody overnight and washed for three times. Subsequently, they were incubated with second antibody in a dark humidity chamber at 4 °C for 1 hand subsequently washed five times with PBS. For immunofluorescence staining of human normal and calcified aortic valve tissue, slices were incubated with anti-IL-21(ab5978, 1:100), anti-IL-21R (ab5980, diluted 1:100), antiCD3 (Abcam, ab699, 1:1000), anti-IL-a-SMA (ab119542, 1:100), overnight, subsequently, they were incubated with second antibody in a dark humidity chamber at 4 °C for 1 hand subsequently washed five times with PBS. Nuclei were stained with DAPI (solarbio, C0060l, diluted 1:5000). Cells and tissue samples were viewed by a fluorescence microscope (Olympus).

### Transfection with siRNA

To inhibit STAT3, IL-21R expression, cultured VICs at 70-80% confluency were transfected with specific siRNA (100 nM) or scramble siRNA (100 nM) using Lipofectamine 3000 (Invitrogen, USA) according to the manufacturer's recommendations. After incubated (at 37 °C and 5% CO_2_) for 6 h, the medium was replaced with DMEM (Hyclone) containing 10% fetal bovine serum (Gibco, Invitrogen). After transfection, the cells were harvested at 48 h for RNA and protein extraction.

### Statistical analyses

Data are expressed as means ± SEM. All experiments were independently replicated in cells from at least three different human aortic valves. Differences between multiple groups were evaluated via ANOVA with post-hoc analysis. *P* < 0.05 was considered to be statistically significant. All statistical analyses were performed using GraphPad Prism software for Windows.

## Results

### IL-21 is expressed at high levels in calcific AV Leaflets

**Figure 1:**
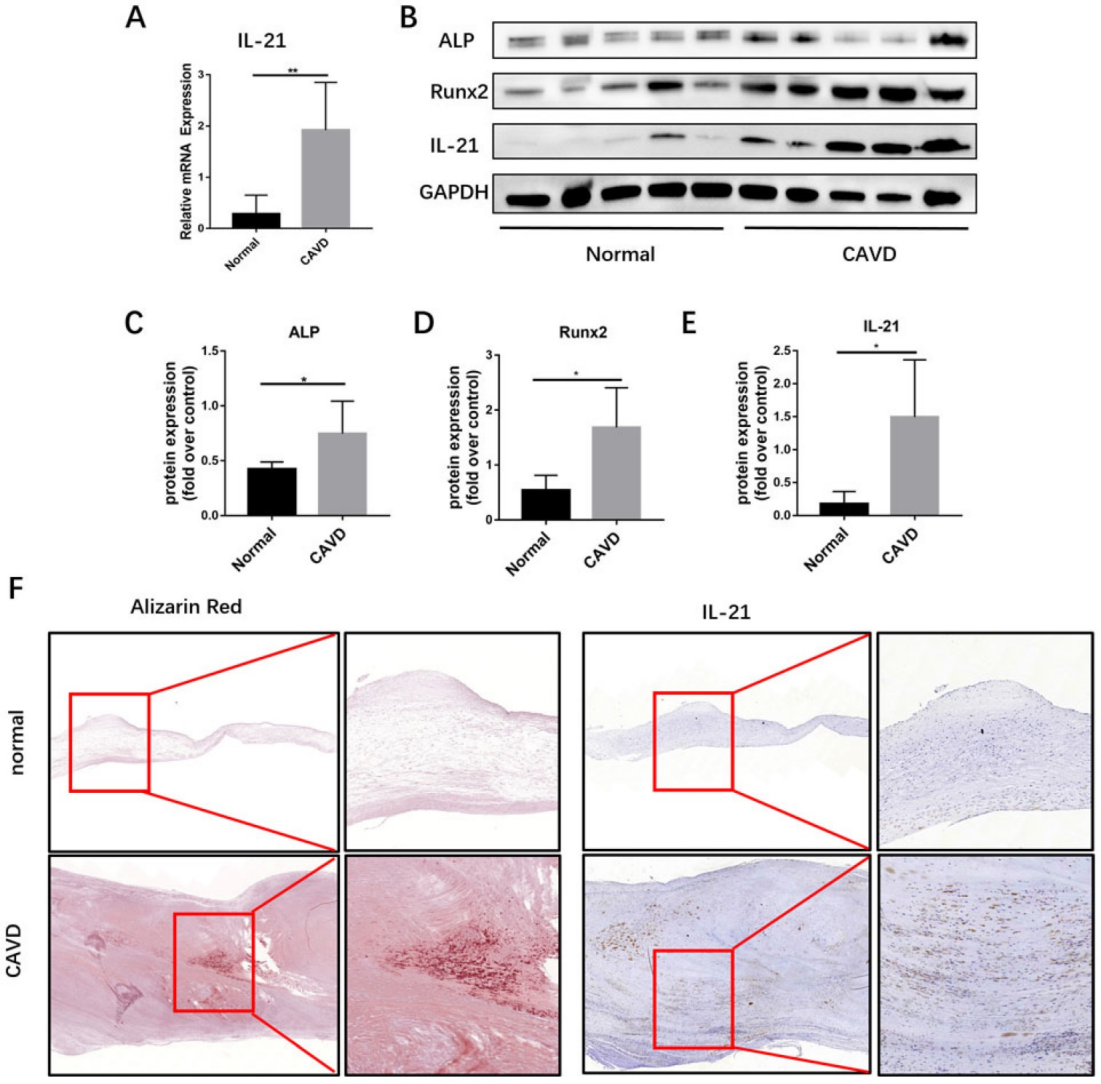
** IL-21 expression in human calcific AVs.** A) Relative mRNA expression. B-E) IL-21, ALP, and Runx2 expression in human calcific AVs. F) Representative images of immunohistochemical staining for IL-21 in (top row, 4X) normal and (bottom row, 8X) calcific AVs; n = 5 for each group. **P < 0.01.

To identify additional cytokines that may promote CAVD pathogenesis, we analyzed *IL-21* mRNA expression in calcific AVs from CAVD (n=5). We used qPCR to measure *IL-21* expression in CAVD Leaflets from CAVD patients and healthy controls (Figure 1A). The immunofluorescence showed that CD3^+^ T cells and IL-21 were co-stained in the CAVD leaflets (Figure S1).

Then, we used immunoblotting and immunohistochemical staining to determine whether IL-21 are involved in calcific AV disease and found that the expression of IL-21 runx2 and ALP were markedly increased in calcific AVs, compared to normal controls (Figure 1, B-E). By immunohistochemistry, regions of IL-21 expression in diseased aortic valve samples were predominantly in areas adjacent to calcifications (Figure 1F). These results suggest that IL-21 plays a role in AV calcification.

### IL-21 promotes osteogenic differentiation of VICs

**Figure 2:**
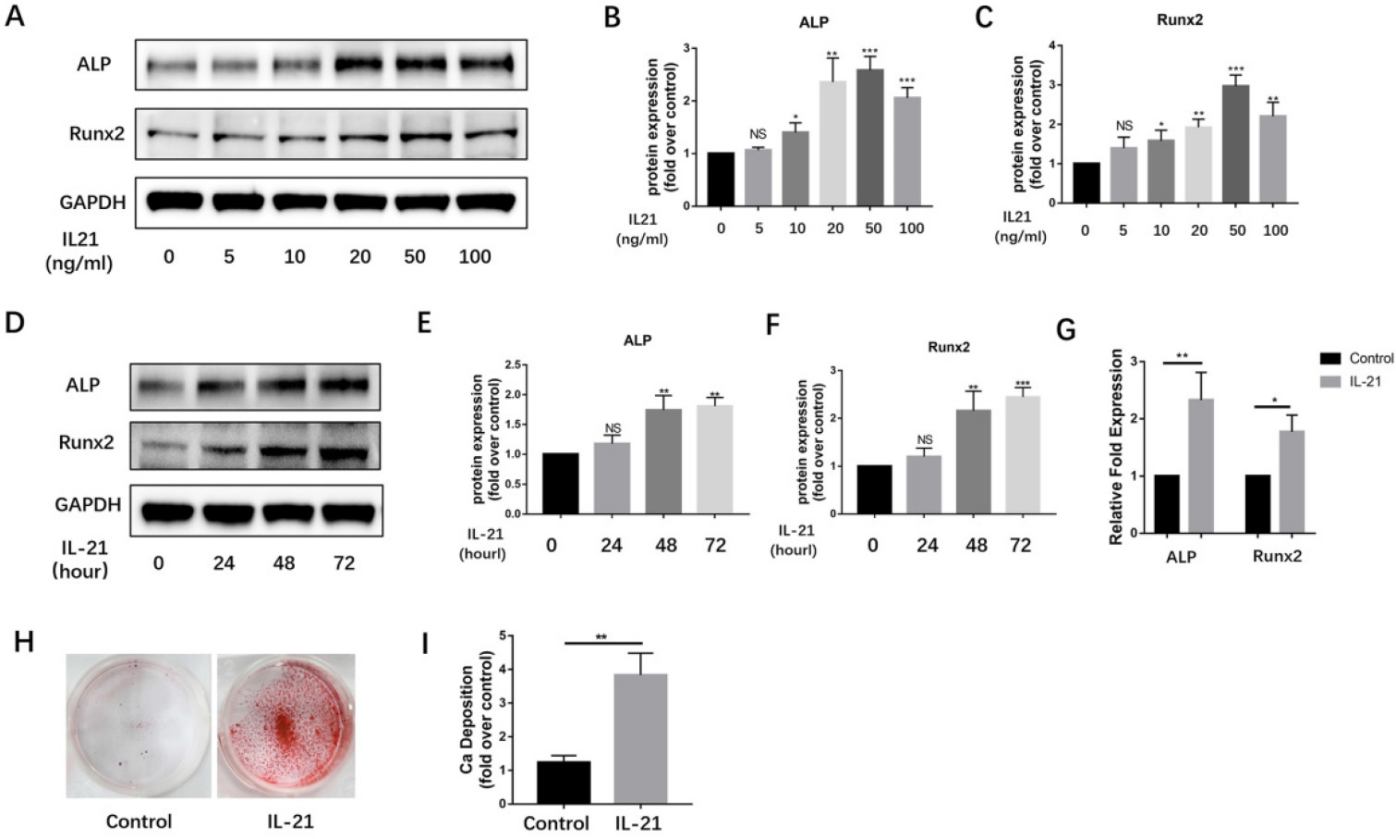
** IL-21 protein induces osteoblastic differentiation of aortic valve interstitial cells.** A-C) ALP and Runx2 expression in VICs (n = 3). D-F) Time course of IL-21 induction of ALP and Runx2 expression in VICs (n = 3). G) Effect of IL-21 (50 ng/ml) treatment on *ALP* and *Runx2* mRNA expression in VICs for 3 days (n = 3). H-I) IL-21 induces deposition of calcium in VICs (n = 3). NS >0.05, *P < 0.05, **P < 0.01, ***P < 0.001.

Over the past decade, VICs have been widely considered to be the major source of osteoblast-like cells in calcified aortic valves. We found IL-21 to be expressed at high levels on the surface of VICs. To determine if IL-21 affects osteogenic differentiation of VICs, we treated VICs with IL-21 at 0.5, 10, 20, 50, and 100 ng/ml. After 3 days of stimulation, IL-21 increased the expression of the osteoblastic markers Runx2 and ALP in a dose-dependent manner (Figure 2A-C). We tested a single concentration of IL-21 (50 ng/ml) on VICs at 0, 24, 48, and 72 h). The expression of Runx2 and ALP increased in a time-dependent manner (Figure 2D-F). Their mRNA expression levels also significantly increased in the presence of IL-21 (50 ng/ml) (Figure 2G). To confirm whether IL-21 stimulation promotes matrix mineralization and calcium deposition in cultured normal VICs, we treated VICs with conditioned medium for 21 days and found a significant increase in calcium deposition (Figure 2H and I).

### IL-21R knockdown inhibits IL-21 mediated osteogenic differentiation of VICs

To verify whether IL-21/IL-21R play central roles in the osteogenic differentiation of VICs. We first examined IL-21R expression in VIC. Using immunofluorescence, IL-21R was found to express in calcified valves especially with the fibroblast markers α-SMA co-expression (Figure S2). During IL-21 stimulation, the in vitro experiment also stated the increased expression of IL-21R compared with the control non IL-21 stimulation group (Figure S3B). We designed siRNA sequences for silencing *IL-21R* gene expression for mechanism exploration. Immunoblotting showed that knockdown of *IL-21R* significantly downregulated expression of IL-21R protein (Figure S2C). Moreover, knockdown of *IL-21R* inhibited the IL-21-induced expression of the osteoblastic markers ALP and Runx2 (Figure S3D, S3E and S3F).

### JAK3 inhibition inhibits osteogenic differentiation

**Figure 3:**
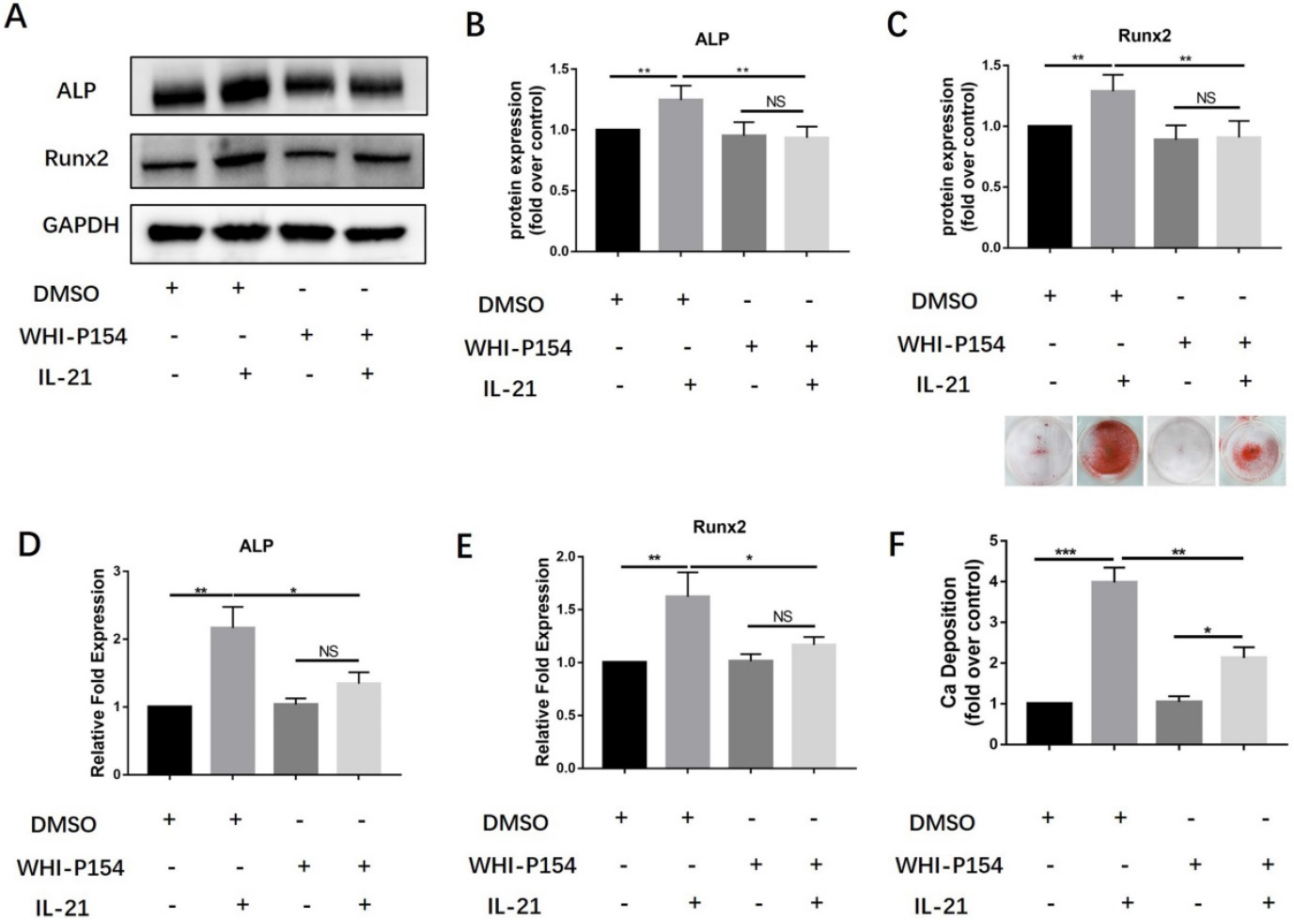
** Effect of JAK3 inhibition on osteogenic differentiation of VICs induced by IL-21.** A-C) Expression of Runx2 and ALP in VICs treated with IL-21 for 3 days, then treated with WHI-P154. D-E) *ALP* and *Runx2* mRNA expression. F) Alizarin Red S staining was used to observe calcium deposition in human AVICs after 21 days in culture with different interventions, NS >0.05, *P < 0.05, **P < 0.01, ***P < 0.001.

Previous studies have shown that the interaction between IL-21 and its receptor can induce activation of Janus kinase-3 (JAK3) and subsequently activate signal transducer and activator of transcription-3 (STAT3). We further studied the roles of the JAK3/STAT3 signaling pathway in the induction of osteoblast differentiation of VICs. We treated them with a specific inhibitor of JAK3, WHI-P154. We found that 10 µM WHI-P154 decreased IL-21-induced expression of the osteoblastic markers Runx2 and ALP (Figure 3A-C), with a concomitant decrease in their mRNA expression (Figure 3D-E). In our study, we found that pre-treatment of VICs with a specific inhibitor of JAK3,10 µM WHI-P154 resulted in reduced protein expression of Runx2 and ALP (Figure 3A-C), with a concomitant decrease in their mRNA expression (Figure 3D-E), and consistently ,decreased matrix calcium deposition after stimulation with IL-21 (50 ng/ml) (Figure 3F).

### *STAT3* knockdown inhibits IL-21-induced osteoblastic differentiation

**Figure 4:**
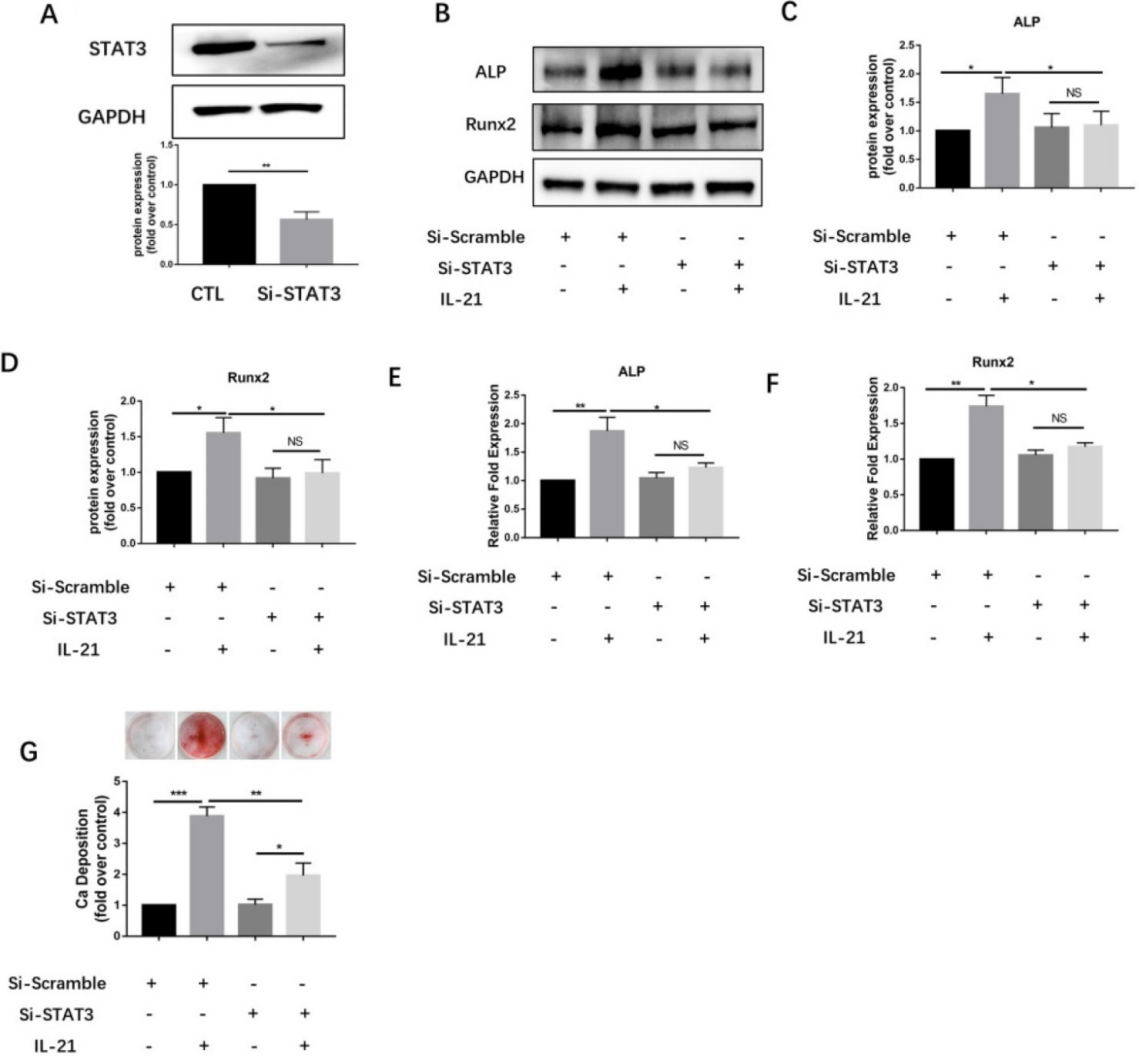
** STAT3 knockdown inhibits IL-21-induced osteoblastic differentiation of VICs.** A) Expression of STAT3 48 h after siRNA introduction. B-D) Western blotting showing inhibition of IL-21-induced expression of ALP and Runx2. E-F) *ALP* and *Runx2* mRNA expression. G) Alizarin Red S staining was used to observe calcium deposition in VICs after 21 days in culture with different interventions. NS >0.05, *P < 0.05, **P < 0.01, ***P < 0.001.

To further validate the JAK3-related STAT subsets, we designed siRNA sequences for silencing *STAT3* gene expression. Immunoblotting showed that knockdown of *STAT3* significantly downregulated expression of STAT3 protein (Figure 4A) and inhibited the IL-21-induced expression of the osteoblastic markers ALP and Runx2 (Figure 4B and D). Meanwhile, knockdown of STAT3 using siRNA significantly suppressed the induction of *ALP* and *Runx2* mRNA in VICs (Figure 4E and F), and consistently, decreased matrix calcium deposition after stimulation with IL-21(50 ng/ml) (Figure 4G).

## Discussion

A large amount of evidence indicates the pathological processes of CAVD share mechanisms with ectopic ossification in cardiovascular system, including inflammation, angiogenesis, and ECM remodeling 18, 19. CAVD is characterized by chronic inflammation of the aortic valve leaflet and infiltration by blood-derived immune cells, which show signs of activation and produce high levels of proinflammatory cytokines, including IL-6, IL-18 and TNF-α 20, 21. Thus, it is important to understand the relationships between CAVD and proinflammatory cytokines.

IL-21 is involved in several inflammatory disorders. Previous research has demonstrated that IL-21, IL-17, IL-23 can lead to severe valve damage 22. The Th2 cytokines IL-4, IL-5, IL-13 and IL-21 each have distinct roles in the regulation of tissue remodeling and fibrosis 23. IL-21 signaling was recently shown to promote fibrosis by facilitating the CD4^+^ Th2 response 24. Therefore, we first found that the expression of IL-21 was markedly increased in calcific AVs. By immunohistochemistry, regions of IL-21 protein expression in diseased aortic valve samples were adjacent to areas of calcification. And IL-21R might also participate in the osteogenic differentiation of VICs according to our results. These results suggested that IL-21/IL-21R axis plays a role in the development of AV calcification.

IL-21 activates multiple signaling pathways, including the Janus kinase 1 and 3-signal transducer and activator of transcription (STAT3) pathway, the MAPK pathway, and the PI3K/AKT signalling pathway 25 through its receptor. Some studies have shown that members of the IL-6 family (IL-6, OSM) regulate osteoblastic differentiation of dental-pulp stem cells, human bone-marrow mesenchymal stem cells, and vascular smooth-muscle cells through the STAT3 pathway 11, 26. STAT3 plays an essential role in heart protection 27. It has been reported that cardiomyocyte-restricted deletion of *Stat3* resulted in decreased myocardial capillary density and increased interstitial fibrosis 28. Runx2 plays a central role in coordinating multiple signals involved in osteoblast differentiation 29. STAT3 can physically interact with Runx2 to impair its transcriptional activity 30. The study have shown that IL-21 enhances both RA-FLS proliferation and their production of IL-6 and TNF-a by STAT3 31. Since RA-FLS shared a similar phenotype with VICs in the context of IL-21R expression, based on these results we can deduce that IL-21 and JAK/STAT3 signaling play important roles in the osteogenetic differentiation of VICs. Further studies on p-STAT3 mediated binding to the *ALP* and *Runx2* promoters are needed to characterize the nuclear localization signal.

So far, a tremendous amount has been learned about the actions of IL‑21 on a broad array of target cells, there are two general areas where the positive effects of IL‑21 are important. First, in cancer immunotherapy there has been a rapid progression from animal studies to clinical trials for several classes of solid tumours. Second, IL‑21-IL‑21R blockade may also have therapeutic benefit in the treatment of autoimmune diseases and inflammatory conditions 25. According to our findings, targeting IL-21/IL-21R and JAK/STAT3 pathway might be an effective therapeutic method for CAVD which needed animal experiment verification.

## Conclusions

In our study, we showed that IL-21 and JAK/STAT3 signaling increase mineralization of VICs, increasing expression of the osteogenic markers ALP and Runx2, which can be blocked by WHI-P154. We conclude from these findings that IL-21 promotes the differentiation of VICs into osteoblastic lineages and increases mineralization. It is hoped that IL-21 can be a potential target for CAVD therapy.

## Supplementary Material

Supplementary figures and tables.

## Figures and Tables

**Figure 5:**
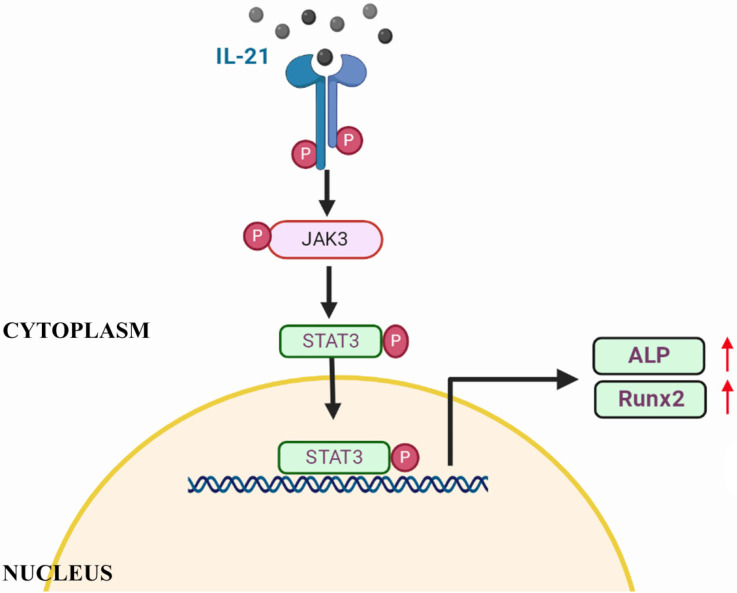
Schematic of IL-21 promotion of osteoblastic differentiation of VICs through the JAK3-STAT3 pathway.
