# Supplementary material for: IL-21 promotes osteoblastic differentiation of human valvular interstitial cells through the JAK3/STAT3 pathway
Source: Int J Med Sci. 2020 Oct 20;17(18):3065–72. doi: 10.7150/ijms.49533 (PMC7646116; doi:10.7150/ijms.49533)
Supplement: Supplementary file 1 — Supplementary figures and tables. [file ijmsv17p3065s1.pdf]

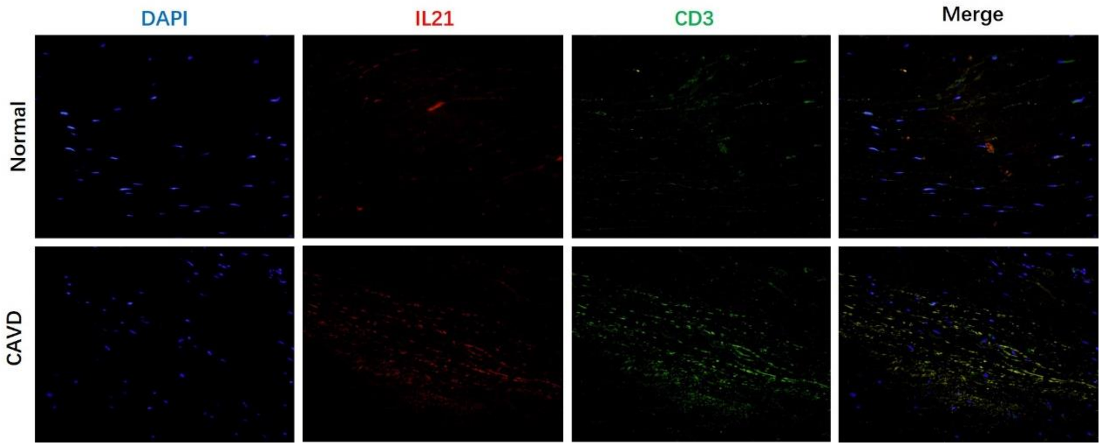

Figure S1 IL-21 expression in human calcific AVs CD3^{+} T cells

Human AV sections analyzed by double-immunofluorescence staining with antibodies against IL-21 (red) and CD3 (greed).DAPI (blue) indicates nuclear staining.

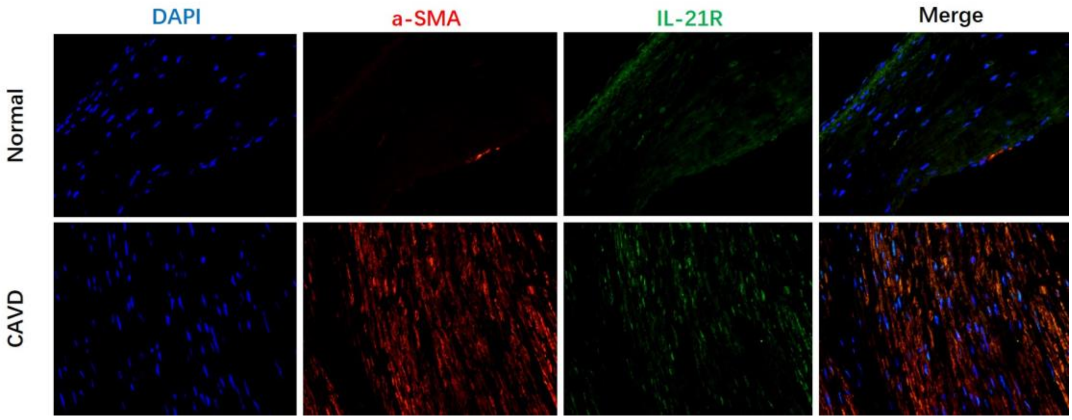

Figure S2 IL-21R expression in human calcific AVs VICs.

Human AV sections analyzed by double-immunofluorescence staining with antibodies against α-SMA (red) and IL-21R (greed).DAPI (blue) indicates nuclear staining.

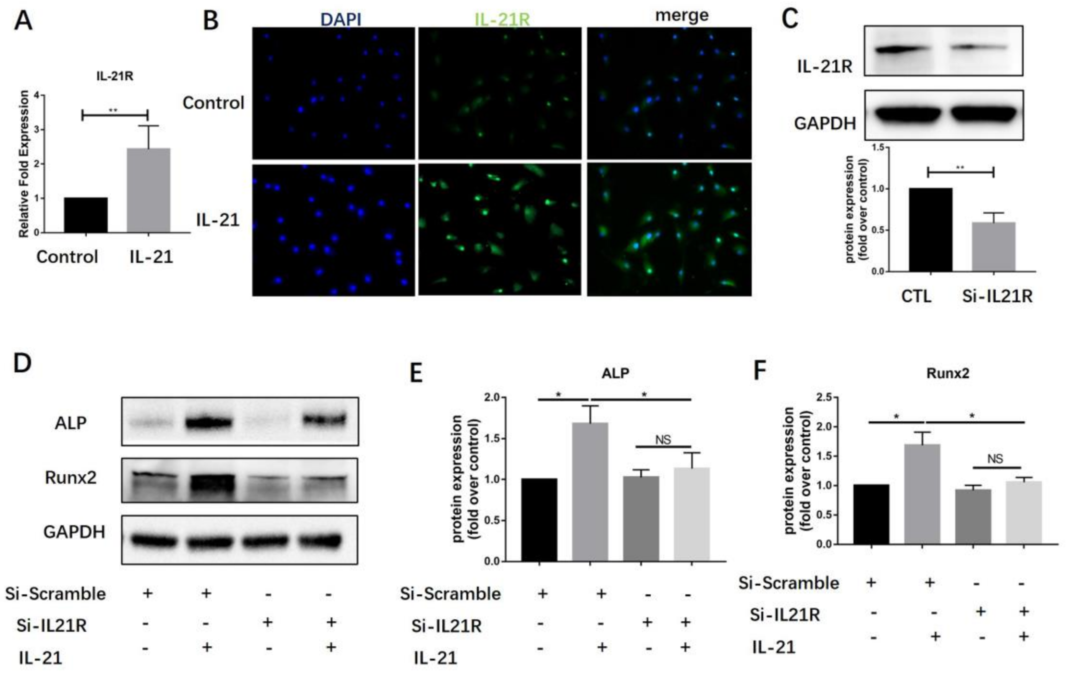

Figure S3 IL-21R knockdown inhibits IL-21-induced osteoblastic differentiation of VICs

A) Effect of IL-21 (50ng/ml) treatment on IL-21R mRNA expression in VICs for 2 days. B) Representative images of immunofluorescent staining for IL-21R in VICs (200X). C) Expression of IL-21R 48 h after siRNA introduction. D-E) Western blotting showing inhibition of IL-21-induced expression of ALP and Runx2. NS >0.05， $*P < 0.05$, $**P < 0.01$, $***P < 0.001$.

Table S1. Clinical characteristics of CAVD and control HC patients.

HC, healthy controls; CAVD, calcific aortic valve disease; NS, nonsignificant; AV,

	HC (n=15)	CAVD (n=20)	*P*
Mean age (years)	43.6 ± 2.1	59.8 ± 1.7	NS
Male/total	7/15	15/20	< 0.05
Weight (kg)	63.9 ± 1.7	68.9 ± 1.4	NS
Hypertension	4/15	13/20	< 0.05
Hypercholesterolemia	4/15	9/20	< 0.05
Diabetes mellitus	3/15	5/20	NS
Smoking	6/15	7/20	NS
statins	3/15	3/20	NS
ACEi/ARB	4/15	5/20	NS
b-blockers	2/15	8/20	< 0.05
LVEF (%)	37.7 ± 5.1	56 ± 2	< 0.05

Aortic valve; ACEi, angiotensin-converting enzyme inhibitor; ARB, angiotensin receptor blocker; LVEF, left ventricular ejection fraction.

Table S2. Sequences (5′-3′) of oligonucleotide primers and probes. R, reverse; F, forward.

Homo RUNX2 F	GGCGGGTAACGATGAAAATT
Homo RUNX2 R	GAGGCGGTCAGAGAACAAACTA
Homo ALP F	GACAAACTGGGGCCTGAGATA
Homo ALP R	CTGACTTCCCTGCTTTCTTGG
homo IL-21 F	AGGTCAAGATCGCCACATGA
homo IL-21 R	TGCTGACTTTAGTTGGGCCT
homo IL-21R F	ACTCTTGGATGCAGGGACC
homo IL-21R R	AAGCCACTGTCAAACGTGTC
Homo GAPDH F	TCAAGAAGGTGGTGAAGCAGG
Homo GAPDH R	TCAAAGGTGGAGGAGTGGGT
